# Evaluation of three patient reported outcome measures following operative fixation of closed ankle fractures

**DOI:** 10.1186/s12891-018-2051-5

**Published:** 2018-05-02

**Authors:** Andrew M. Garratt, Markus G. Naumann, Ulf Sigurdsen, Stein Erik Utvåg, Knut Stavem

**Affiliations:** 1 Division for Health Services, Norwegian Institute of Public Health, Oslo, Norway; 2 Department of Orthopaedics, Østfold Hospital, Oslo, Norway; 3 Department of Orthopaedics, Akershus University Hospital, Lørenskog, Norway; 4 Institute of Clinical Medicine, University of Oslo, Oslo, Norway; 5 Department of Pulmonary Medicine, Medical Division, Akershus University Hospital, Lørenskog, Norway; 6 Health Services Research Unit, Akershus University Hospital, Lørenskog, Norway

**Keywords:** Ankle, Patient reported outcome measures, PROMs, PROs, Validity, Reliability

## Abstract

**Background:**

Several patient reported outcome measures (PROMs) are available for assessing the outcomes of ankle fracture but few have been compared for recommended measurement properties. This study compares the measurement properties of the Lower Extremity Function Scale (LEFS), Olerud Molander Ankle Score (OMAS) and Self-Reported Foot and Ankle Score (SEFAS) following ankle surgery.

**Methods:**

The retrospective cohort study included 959 patients aged 18 years and over who underwent surgical treatment (ORIF) for unstable and closed ankle fractures in SE Norway. The PROMs were included in a postal questionnaire sent to patients’ homes in 2015, three years after surgery. Missing data, structural validity, internal consistency, test-retest reliability and validity were assessed.

**Results:**

Confirmatory factor analysis results showed model fit for the SEFAS and a bi-dimensional LEFS with scales of easy and difficult items. The OMAS performed less satisfactorily. Cronbach’s alpha and test-retest correlations ranged from 0.82 to 0.96 and 0.91 to 0.93 respectively. The smallest detectable differences for group and individual comparisons were 14.1 to 20.6 and 0.93 to 1.55; SEFAS performed best. As hypothesised, instrument scores were highly correlated and with those for the EQ-5D and SF-36 physical functioning. Mean imputation where half or more items are completed increased usable scores by 1.4–15.7% without affecting measurement properties.

**Conclusions:**

The three instruments largely performed satisfactorily in relation to important measurement properties but the LEFS had evidence for two dimensions relating to easier and more difficult aspects of function. Mean imputation where half or more items are completed increased the number of usable responses for all three instruments. The three instruments represent different approaches to measuring outcomes and their content should be considered carefully when choosing between them. The SEFAS is designed for a range of foot disorders including ankle fractures and has the best measurement properties in this population.

## Background

Ankle fracture constitutes approximately 9% of all fractures, have an incidence of 122 per 100,000 people [1] and incidence requiring hospitalisation of 83 per 100,000 people [2]. Following a systematic review it was concluded that there was insufficient evidence as to whether conservative management or surgery gives the best long-term outcomes in adult patients [1]. Moreover, the evidence derived from a systematic review of competing surgical technologies led to the conclusion that further evaluation, including greater consideration of long-term outcomes, was necessary [3].

Ankle fractures reduce quality of life and particularly in older people, may cause loss of independence. It is important that studies evaluating outcomes in these patients include valid and reliable patient reported outcome measures (PROMs) that reflect important concerns of patients [4]. There are a large number of ankle-specific PROMs [5] but few have been developed with the input of fracture patients or sufficiently evaluated for measurement properties [4]. Clinicians and researchers wishing to select an ankle specific PROM are faced with a confusing array of instruments with little information on measurement properties in ankle fracture patients. When a choice of instrument exists, the concurrent evaluation of their measurement properties in the patients and health care setting of interest is highly informative [6]. Systematic reviews of measurement properties are also informative, however, the two published reviews focus on ankle problems more generally [5] or ligament injuries [7].

The Lower Extremity Functional Scale (LEFS) [8], Olerud and Molander Ankle Score (OMAS) [9] and Self-Reported Foot and Ankle Score (SEFAS) [10] have been widely applied but have undergone limited testing for measurement properties in patients with ankle fractures. These three instruments represent different approaches to measuring outcomes in patients with ankle fractures; the LEFS focuses on lower limb, the OMAS is ankle fracture specific, and the SEFAS is foot and ankle specific. None of them have been tested for structural validity, which gives evidence supporting their scoring as unidimensional scales. This study compares important measurement properties of these instruments, including reliability and validity [11].

## Methods

### Study population

The retrospective cohort study included 959 patients who underwent surgical treatment (ORIF) for unstable and closed ankle fractures at two hospitals in SE Norway [12]. Patients were 18 years of age and over and treated in a three year period from January 1, 2009. They received a postal questionnaire that included LEFS, OMAS and SEFAS in January 2015; 299 respondents received a test-retest questionnaire at six weeks. Non-respondents received a reminder at four weeks.

### Patient-reported outcome measures

Norwegian translation of the three instruments followed international guidelines [11] including two independent forwards and one independent backwards translations with a meeting to agree on the final Norwegian versions.

The LEFS comprises 20 items relating to physical function and daily activities with a five-point scale from ‘extreme difficulty or unable to perform’ to ‘no difficulty’ [8]. Items are summed to give a score from 0 to 80 where 80 is the best possible score. The mean of the completed items is used when up to four items are missing [8, 13] and normative data is available to aid the interpretation of LEFS scores [14]. Two studies have assessed the measurement properties of LEFS in patients with ankle fracture and there is evidence for reliability, validity and responsiveness in Australian patients [15] and in Finnish patients undergoing surgery due to musculoskeletal pathology of the foot and ankle [16].

The OMAS comprises nine items relating to symptoms, physical function and daily activities [9]. The response scales vary from binary to five-points with clinical scoring that reflects the level of disability for individual items. Item responses are summed to give a score from 0 to 100 with higher scores representing the best possible. The instrument has evidence for test-retest reliability and construct validity in patients with ankle fracture in Sweden [17] and Turkey [18].

The SEFAS comprises twelve items relating to pain, limping, swelling, use of orthotics and walking. The five-point scales reflect item content and sum to give a score from 12 to 60 where the former represents normal function [10, 19]. The mean of the completed items is used when one item is missing. The instrument has not been evaluated solely in patients with ankle fracture but has evidence reliability, validity and responsiveness in Swedish patients with foot and ankle disorders undergoing surgery [10, 19]. For purposes of comparison, scores for the LEFS and SEFAS are also presented on a 0 to 100 scale where higher scores represent the best possible.

Two generic instruments were also included in the questionnaire. The EQ-5D-3L includes five items with a three-point response scale which are scored to give a single index [20]. The SF-36 physical function scale comprises ten items with a three-point scale which sum to a 0 to 100 scale where 100 is the best possible health [21].

### Statistical analysis

The measurement properties tested and related terminology follow the COSMIN checklist [11]. Levels of missing data were assessed at the item and scale level with the latter also including imputation for missing data where half or more item responses were present. For comparison, all items were recoded from 0 to 4 where 4 is the best possible health.

Confirmatory factor analysis (CFA) with weighted least squares estimation was used to assess structural validity. Model fit was assessed with the comparative fit index (CFI), Tucker-Lewis Index (TLI) and root mean square error of approximation (RMSEA) [22–24]. The CFI and TLI should be greater than 0.90 and RMSEA between 0.06 and 0.08 for acceptable fit [24, 25]. Internal consistency was assessed using item-total correlation which should exceed 0.4 and Cronbach’s alpha, which should exceed 0.7 and 0.9 for use in groups and individual patients, respectively [26]. The intraclass correlation coefficient was used for estimating reliability within a two-way mixed effects model with absolute agreement. Weighted kappa was used for assessing individual item reliability [27]. The standard error of measurement (SEM) and smallest detectable change (SDC) were calculated. The former is the square root of the total error variance. For individuals the SDC is 1.96 × √2 × SEM and for groups, the SDC for individuals is divided by √n [26].

Hypothesis testing was used to assess the validity of the three ankle instrument scores through comparisons of those for the EQ-5D and SF-36 physical functioning and clinical variables. These instruments were included in previous tests of validity for the LEFS [13] and SEFAS [10] and continue to be the most widely evaluated and applied PROMs [6]. It was hypothesised that scores for the three instruments would be highly correlated over 0.7. High levels of correlation were expected with SF-36 physical functioning and particularly for the LEFS, given the overlap in content. The three instruments include items that overlap with two or more EQ-5D items and hence high levels of correlation were expected for EQ-5D scores and the EQ-5D mobility item. Moderate levels of correlation in the range 0.5 to 0.7 were expected for the EQ-5D usual activities and pain items. Lower levels of correlation in the range 0.3 to 0.5 were expected for the EQ-5D self-care and anxiety/depression items. Lower levels of correlation under 0.3 were hypothesised for the clinical variables including ASA classification, BMI, duration of operation and fracture classification.

LISREL was used for the CFA and PASW Statistics 18.0 was used for the remainder of the statistical analysis.

## Results

### Study population

The questionnaire was returned by 567 (59.1%) patients. Table 1 shows the characteristics of respondents. There were 182 (60.9%) respondents to the test-retest questionnaire.

**Table 1:** Characteristics of respondents completing the first questionnaire (*n* = 567)

		%
Age years, mean (range)	57.5 (22.2–91.2)	
Female	322	56.8
Marital status^a^		
Divorced/separated	77	13.6
Cohabitant/married	379	66.8
Single	47	8.3
Widowed	46	8.1
Education		
Under 10 yrs	157	27.7
10–12 yrs	206	36.3
University	181	31.9
Body mass index (kg/m^2^), median range	27.4 (14.4–61.0)	
Current smoker	136	25.2
Diabetes	34	6.0
Fracture classification, Weber		
A	15	2.6
B	383	67.5
C	156	27.5
Fracture, clinical features		
Uni-malleolar	294	52.5
Bimalleolar	128	22.9
Trimalleolar	138	24.6
Physical status (ASA classification)		
Completely healthy fit	197	34.7
Mild systemic disease	340	60.0
Severe systemic disease, not incapacitating	30	5.3
Postoperative length of stay in days, median (range)		
Surgery duration in minutes, median (range)	76.0 (7–352)	
Waiting time for surgery in days, median (range)	5.0 (0–68)	

^a^Percentages do not sum to 100 due to missing data

### Statistical analysis

Levels of missing data ranged from 1.2 to 6.2% across the three instruments (Table 2). Levels of missing data were highest for items assessing higher levels of function including ‘hopping’ and ‘running’ for LEFS and ‘jumping’ for OMAS. For the LEFS, the ‘getting in and out of bath’ had the highest level of missing data. Use of mean imputation for missing data increased the number of useable scores by 1.4, 6.9 and 15.7% for the LEFS, SEFAS, and OMAS respectively (Table 2).

**Table 2:** Descriptive statistics, internal consistency (*n* = 567) and reliability (*n* = 182) where all items are coded^a^ 0 to 4 for comparison

Instrument/item	% miss	Mean (SD)	Frequencies %					Cronbach’s alpha (scale)/item scale correlation	Test-retest ICC (scores), weighted kappa (items)
			0	1	2	3	4		
Lower Extremity Functional Scale LEFS^b^	4.2	67.20 (15.09)						0.96	0.91
LEFS adjusted^c^	2.8	83.89 (19.01)						–	0.91
1 Usual work, housework, school	2.6	3.51 (0.89)	1.8	2.5	8.2	17.5	70.0	0.81	0.77
2 Hobbies, recreational, sports	4.9	3.22 (1.02)	2.8	5.4	10.2	29.8	51.8	0.78	0.76
3 Getting in and out of bath	5.8	3.54 (0.97)	3.4	3.2	5.1	12.4	75.9	0.72	0.76
4 Walking between rooms	2.8	3.91 (0.40)	0.2	0.5	0.9	5.3	93.1	0.56	0.70
5 Putting on socks or shoes	3.0	3.65 (0.72)	0.9	1.5	4.9	17.3	75.4	0.70	0.71
6 Squatting	3.0	3.03 (1.30)	7.8	9.1	8.0	21.7	53.3	0.73	0.74
7 Lifting things up from floor	2.1	3.82 (0.57)	0.9	0.5	2.2	8.0	88.4	0.61	0.79
8 Light activities in the home	2.5	3.82 (0.54)	0.2	0.9	3.1	8.5	87.3	0.69	0.78
9 Heavy activities in the home	2.5	3.27 (1.05)	2.7	6.5	9.4	23.2	58.1	0.85	0.85
10 Getting in/out of car	2.5	3.72 (0.64)	0.2	1.6	4.4	14.2	79.7	0.67	0.68
11 Walking 2 blocks	3.0	3.69 (0.82)	2.4	1.5	4.4	8.2	83.6	0.74	0.80
12 Walking a mile	3.4	3.42 (1.07)	4.2	3.8	7.9	13.7	70.3	0.82	0.84
13 Going up or down ten stairs	3.2	3.65 (0.78)	1.1	2.4	4.9	13.9	77.7	0.75	0.73
14 Standing 1 h	2.8	3.11 (1.21)	6.0	7.8	8.6	24.0	53.6	0.84	0.85
15 Sitting 1 h	3.4	3.77 (0.65)	0.5	1.8	3.1	9.5	84.8	0.51	0.57
16 Running on even ground	5.1	2.74 (1.46)	13.6	10.3	9.9	20.7	45.5	0.85	0.87
17 Running on uneven ground	4.4	2.46 (1.47)	16.3	12.8	13.0	24.4	33.5	0.83	0.85
18 Sharp turns when running fast	4.9	2.53 (1.52)	17.1	12.5	9.3	22.0	39.1	0.84	0.82
19 Hopping	4.6	2.43 (1.53)	18.2	13.2	12.2	19.9	36.5	0.83	0.83
20 Rolling over in bed	2.7	3.82 (0.54)	0.4	1.1	1.6	10.0	86.7	0.56	0.65
Olerud-Molander Ankle Score OMAS	17.3	74.12 (24.91)						0.82	0.92
OMAS adjusted^c^	1.6	75.62 (24.07)						–	0.91
1 Pain when walking	4.9	3.01 (1.21)	7.3	4.8	13.8	27.6	46.6	0.67	0.76
2 Stiffness (2 pt. scale)	2.7	1.81 (1.99)	54.7				45.3	0.55	0.77
3 Swelling (3 pt. scale)	3.5	2.83 (1.53)	17.1		24.2		58.7	0.51	0.80
4 Use of stairs (2 pt. scale)	1.2	3.25 (1.03)	1.4		35.8		63.8	0.73	0.81
5 Running (2 pt. scale)	2.5	2.66 (1.89)	33.6				66.4	0.66	0.81
6 Jumping (2 pt. scale)	6.2	2.60 (1.91)	34.9				65.1	0.65	0.80
7 Squatting (2 pt. scale)	4.6	2.68 (1.88)	33.0				67.0	0.61	0.68
8 Assistive devices (3 pt. scale)	3.2	3.72 (0.91)	4.4		5.3		90.3	0.37	0.90
			*0*	*1.33*		*2.67*	*4*		
9 Work and daily activities (4 pt. scale)	2.5	3.27 (1.14)	6.4	5.1		25.8	62.8	0.66	0.77
Self-Reported Foot & Ankle Score SEFAS	8.5	20.78 (9.16)						0.93	0.93
SEFAS adjusted^c^	1.6	81.40 (19.17)						–	0.93
1 Usual pain level	1.6	2.76 (1.51)	2.9	14.9	19.1	29.3	33.8	0.82	0.83
2 Walking time before pain too much	3.7	3.58 (0.83)	0.4	5.0	5.1	14.7	74.8	0.69	0.76
3 Work not done as carefully as usual	2.5	3.27 (0.96)	2.2	3.8	12.0	28.5	53.5	0.69	0.78
4 Use of special innersoles and shoes	1.9	3.54 (1.06)	4.7	4.2	2.7	8.8	79.6	0.35	0.69
5 Usual work, housework, hobbies	2.1	3.29 (0.99)	1.1	6.9	11.0	23.5	57.5	0.87	0.85
6 Limping	1.6	3.02 (1.23)	6.8	4.5	22.7	11.0	55.0	0.75	0.77
7 Staircase	2.3	3.55 (0.80)	0.9	2.0	8.3	18.5	70.3	0.71	0.80
8 Problem in bed	1.6	3.21 (1.10)	2.5	4.7	22.7	10.1	60.1	0.72	0.70
9 Usual free time activities	1.8	3.11 (1.09)	2.7	9.2	10.6	29.7	47.7	0.86	0.88
10 Swelling	2.1	3.00 (1.20)	7.1	6.9	9.4	32.4	44.3	0.57	0.90
11 Getting up from a chair	1.8	3.60 (0.72)	0.2	1.8	7.0	19.6	71.4	0.77	0.77
12 Sudden strong pain	1.8	3.15 (1.02)	1.3	3.4	27.7	14.2	53.2	0.68	0.77

^a^To aid comparison, all items were coded from 0 to 4 where 4 is the best possible health; OMAS values in italics represent the four scale points for item 9 converted to the 0–4 scale. LEFS and SEFAS have five-point scales for all items. OMAS scaling varies across items which gives the blank spaces for those items with four or less scale categories

^b^Instrument scoring: LEFS 0–80 where 80 is the best possible; LEFS adjusted 0–100 where 100 is the best possible; OMAS 0–100 where 100 is the best possible health; SEFAS 12–60 where 60 is the worst possible; SEFAS adjusted 0–100 where 100 is the best possible health

^c^Mean imputation was used when half or fewer items were missing

Item mean scores were mostly skewed towards the best possible scores across instruments (Table 2). For the LEFS, the lowest scores denoting poorer health were for ‘hopping’ and the highest scores were for ‘walking between rooms’. For the OMAS, the lowest scores were for ‘stiffness’ and the highest scores were for ‘assistive devices’. For the SEFAS the highest scores were for ‘getting up from a chair’ and the lowest scores were for ‘usual pain level’.

Model fit for the unidimensional SEFAS was good according to all criteria (Table 3). The LEFS and OMAS had a RMSEA that was over the criterion of 0.08. There was support for a bi-dimensional LEFS with scales relating to easy and difficult items. Item-total correlations were over 0.4 for all items with the exceptions of ‘assistive devices’ and ‘use of special innersoles/shoes’ for the OMAS and SEFAS respectively (Table 1). Cronbach’s alpha ranged from 0.82 to 0.96 for the OMAS and LEFS respectively.

**Table 3:** Confirmatory factor analysis tests and goodness of fit indices (*n* = 567)

Instrument	χ2	*df*	CFI^a^	TLI^b^	RMSEA^c^
LEFS unidimensional	843.4	171	0.99	0.99	0.091
LEFS bidimensional^d^	604.4	169	1.00	1.00	0.073
SEFAS	165.9	54	0.99	0.99	0.063
OMAS	122.8	27	0.99	0.98	0.087

All χ2 tests of model fit were significant with the exception of the LEFS (*p* < 0.01)

^a^Comparative fit index

^b^Tucker-Lewis Index

^c^Root mean square error of approximation

^d^LEFS bi-dimensional easy (1,3–5,7,8,10,11,13,15,20) and difficult (2,6,9,12,14,16–19) items

Table 4 shows that there were small but insignificant (*p* < 0.05) score improvements across instruments at retest. Weighted kappa for the individual items indicated good agreement between test and retest (Table 2). Intraclass correlations for the scale scores ranged from 0.91 to 0.93 for the LEFS and SEFAS respectively. The use of alternate scoring had little or no effect on ICC levels (Table 2). The SEMs for the adjusted scores which are comparable, ranged from 5.1 to 7.4 for the SEFAS and OMAS respectively (Table 4). The SDC for comparisons of individual patients ranged from 14.1 to 20.6 for the SEFAS and OMAS respectively. The SDC for comparisons of groups of patients ranged from 0.93 to 1.55 for the SEFAS and OMAS respectively.

**Table 4:** Standard error of measurement (SEM) and smallest detectable change (SDC) for the three instruments^a^ (*n* = 182)

Instrument	*n*	Test mean (SD)	Retest mean (SD)	SEM^b^	SDC^c^individual	SDC^c^group
LEFS (0–80)^c^	179	68.54 (15.03)	69.03 (15.14)	4.50	12.49	0.93
LEFS adjusted	181	85.48 (18.39)	86.19 (18.41)	5.61	15.55	1.16
SEFAS (12–60)^b^	172	19.70 (8.99)	19.49 (9.23)	2.39	6.62	0.50
SEFAS adjusted	182	83.85 (18.52)	84.11 (19.01)	5.09	14.10	0.93
OMAS	140	78.04 (24.44)	79.64 (24.29)	6.87	19.04	0.92
OMAS adjusted	176	77.66 (24.38)	78.85 (23.90)	7.41	20.55	1.55

^a^Instruments scored 0–100 unless otherwise stated

^b^Standard error of measurement

^c^Smallest detectable change

^b^Included for comparison with existing studies that have used the same scoring

**Table 5:** Spearman correlations^a^ between the ankle instrument scores, those for generic instruments and clinical variables (*n* = 567)

Instrument	LEFS	LEFS Adjusted^b^	OMAS	OMAS Adjusted	SEFAS	SEFAS Adjusted
OMAS	0.86	0.86				
OMAS Adjusted	0.86	0.86				
SEFAS	−0.84	−0.84	− 0.89	−0.89		
SEFAS Adjusted	−0.84	−0.84	− 0.88	−0.88		
SF-36 physical function	0.85	0.86	0.76	0.77	−0.74	−0.73
EQ-5D index	0.73	0.73	0.79	0.79	−0.80	−0.79
Mobility	−0.65	−0.64	− 0.66	−0.67	0.66	0.66
Self-care	−0.35	−0.36	− 0.32	−0.33	0.34	0.34
Usual activities	−0.60	−0.61	− 0.59	−0.60	0.62	0.62
Pain/discomfort	−0.64	−0.64	− 0.73	−0.73	0.76	0.75
Anxiety/depression	−0.30	−0.31	− 0.30	−0.31	0.31	0.30
ASA classification	−0.26	−0.26	− 0.19	−0.21	0.21	0.20
Body Mass Index	−0.15	−0.16	− 0.21	−0.19	0.14	0.15
Duration of operation	−0.24	−0.23	− 0.25	−0.26	0.24	0.22
Uni-, bi-, tri-malleolar	−0.23	−0.22	− 0.21	−0.23	0.21	0.19

^a^All correlations are significant (*p* < 0.01)

^b^Adjusted scores where mean imputation is used for missing data when half or more items are completed

Table 5 shows that the hypotheses used in validity testing were largely met but some correlations were higher than expected. The lowest correlation between the three ankle instruments was 0.84 (LEFS and SEFAS) and the highest was 0.89 (SEFAS and OMAS). High levels of correlation were found for SF-36 physical functioning scores, the highest being for the LEFS which were comparable to those between the LEFS and other specific instruments. Moderate to high levels of correlation were found for the EQ-5D mobility and pain/discomfort items. For the three instruments, the correlations with the EQ-5D usual activities item were of a similar moderate level and for the remaining two items of self-care and anxiety/depression, of a similar low level. Correlations with the clinical variables were all of a low level, the lowest were for BMI and the highest were for the duration of operation. The use of adjusted scores had very little effect on the size of the correlations.

## Discussion

There was evidence that the LEFS might be bi-dimensional in this group of patients which contrasts with it is use in applications as a unidimensional measure of lower extremity function. Exploratory factor analysis (data not shown) showed that the items loaded onto two clearly discernible factors relating to easier and more difficult aspects of function which gave better results in the CFA. The LEFS with 20 items, is a good deal longer than the OMAS and SEFAS and such a lengthy instrument that assesses one aspect of health is unusual for PROMs. The OMAS and SEFAS are shorter, have acceptable levels of internal consistency, test-retest reliability and the SEFAS has a lower SEM and hence is more capable of measuring change in individuals and groups of patients.

The current study followed previous studies in treating the LEFS as unidimensional in other aspects of testing but results should be treated with caution until further evidence becomes available. This study is a long-term follow-up of patients and the evidence may be different for patients in the shorter-term post-surgery. LEFS items may differ in their relevance in these patients. For example, more difficult items including ‘running’, ‘squatting’ and ‘walking a mile’ might have greater relevance at follow-up as shown by their much lower ceiling effects compared to the remaining items. The inclusion of easier items in the same scale might mask important effects at follow-up. Eighty percent or more patients had the best possible score on seven LEFS items compared to just one item in each of the OMAS and SEFAS. If long-term outcomes are the focus, then these two instruments might be more responsive to change than the unidimensional LEFS. It is recommended that future studies compare the responsiveness to change of these instruments, including treating the LEFS as a bi-dimensional instrument where evidence supports.

There were low levels of missing data at the item level with very few items having more than 5 % missing. These items tended to relate to more difficult activities undertaken less frequently. Hence, the levels of missing data may reflect uncertainty on the part of the patients regarding their performance, or that they may have held back from undertaking such activities due to concerns about the ankle. Such items include running for the LEFS and jumping for the OMAS. The LEFS item ‘getting in and out of bath’ denotes low levels of function and had relatively high levels of missing data which may because many Norwegians do not have a bathtub at home.

All items with the exception of the assistive devices items for the OMAS and special innersoles/shoes in the SEFAS, had acceptable item-total correlations. This indicates that these two items might not be adequately contributing to the construct being measured. For example, patients might be using assistive devices, innersoles and shoes for reasons other than the severity of their ankle problem or because of other health problems. These items might be considered for removal if future studies also find that they make a limited contribution in a similar patient population. Cronbach’s alpha and test-retest correlations were acceptable for the three instruments. Alpha is dependent on the number of items and hence the highest level was expected for the LEFS.

Scores for the three instruments were highly correlated which is evidence that they are assessing very similar aspects of health and have convergent validity. The highest correlations were found between scores for the OMAS and SEFAS which reflects their ankle specific focus compared to the focus on lower extremity function of the LEFS. These two instruments also had slightly higher correlations with the EQ-5D, including individual EQ-5D items. The LEFS had the highest correlations with SF-36 physical functioning scores and several LEFS items that are not covered by the OMAS and SEFAS, have similar content to this SF-36 scale.

For the LEFS, Cronbach’s alpha, test-retest reliability correlation coefficient and the SEM were similar to those previously reported [16]. The OMAS had a slightly higher Cronbach’s alpha than in the previous study [17]. The test-retest reliability correlation coefficient was slightly lower and SEM slightly higher than those previously reported [17]. It follows that the smallest detectable change was larger; 19–20 compared to 16 [17]. The SEFAS had a higher alpha and similar level of test-retest reliability compared to the Swedish study that included patients with hind foot and ankle disorders [10]. The SEM and SDC were not reported in this study.

Recommendations for handling missing data were not available for the OMAS. The conventional approach is mean imputation when half or less items are missing. Compared to the approach that has been recommended for the SEFAS [10], this form of imputation increases the number of patients with final scores by 7%. This reduces sample sizes required in evaluative studies including clinical trials. Mean scores and the results of testing were very similar irrespective of the methods of handling missing data. For example, levels of correlation with the EQ-5D scores were virtually unchanged. The conventional approach will reduce sample size requirements in clinical trials and based on these study findings, will increase useable scores by up to 16% for the OMAS.

Clinicians and researchers selecting PROMs for this group of patients should consider using the SEFAS in preference to the LEFS and OMAS. There is uncertainty surrounding the structural validity of the LEFS, it has greater respondent burden and a broader focus on lower limbs rather than the foot and ankle. The broader focus was reflected in correlations between the LEFS and SF-36 physical function scores which were higher than those between the LEFS, OMAS and SEFAS scores. The OMAS has more complex scoring, performed less satisfactorily than the SEFAS in terms of structural validity and had a higher SEM. The use of mean imputation where half or more items are completed, reduces the number of patients needed for recruitment with negligible effects on measurement properties.

### Study limitations

Important limitations include the follow-up period, potential respondent bias, choice of instruments and lack of testing for other measurement properties. The median time between surgery and questionnaire completion was 4.3 (IQR 3.9–5.1) years [12], and it is important that the measurement properties of the three instruments are assessed at other clinically important follow-up periods. This limitation means that it was not possible to recommend modifications to the instruments including the use of a bi-dimensional LEFS and removal of items across the instruments. The 59% response rate to the questionnaire is acceptable for this type of study but there were some statistically significant differences between respondents and non-respondents to the questionnaire [12]. Other instruments are available that have undergone limited testing in patients with ankle fracture [5], but respondent burden meant that only three instruments could be included in this study. The design of the study also meant that instrument responsiveness to change could not be assessed. This is an important criterion which further aids the selection of instruments for evaluative studies including clinical trials [11].

Assessment of the SEM and SDC followed the COSMIN checklist [11] and have been previously reported for the LEFS and OMAS [16, 17]. The SDC is the level of change that can be considered real change above measurement error and does not consider whether the change is important. The minimal clinically important difference (MCID) or minimal important change (MIC), are levels of change that patients consider important and further help score interpretation [11]. The MIC has not been reported for the study instruments in this patient population and assessment was not possible within the current study design. It is recommended that the MIC be reported in future studies.

## Conclusion

This is the first study that has concurrently evaluated these instruments in patients following surgery for ankle fracture. Moreover, the LEFS and SEFAS have not been previously evaluated solely in patients with ankle fracture. The three instruments have acceptable evidence for internal consistency, test-retest reliability and construct validity. However, there are some doubts about the unidimensionality of the LEFS in this population and it has a relatively large number of items with the largest ceiling effects representing the highest level of functioning. Further testing of these instruments is recommended in patients with ankle fracture including shorter-term follow-up following surgery. Responsiveness to changes in health should also be assessed with instrument completion taking place before and after an intervention of known efficacy. Instrument content should be carefully considered when choosing between these three instruments. The LEFS is specific to the lower extremities and includes a relatively large number of items. The OMAS is designed to be ankle fracture specific and includes clinical weightings whereas the other two instruments are based on simple summed scales. The SEFAS is designed for a range of foot disorders including ankle fractures and has the best measurement properties in this population. Finally, it is recommended that mean imputation is used for missing responses when half or more items are completed by patients.

## Abbreviations

BMI: Body Mass Index
CFA: Confirmatory factor analysis
CFI: Comparative fit index
ICC: Intraclass correlation coefficient
LEFS: Lower Extremity Function Scale
MIC: Minimal Important Change
OMAS: Olerud Molander Ankle Score
ORIF: Open reduction internal fixation
PROMs: Patient reported outcome measures
RMSEA: Root mean square error of approximation
SDC: Smallest detectable change
SEFAS: Self-Reported Foot and Ankle Score
SEM: Standard error of measurement
TLI: Tucker-Lewis Index
